# Arginase activity mediates reversible T cell hyporesponsiveness in human pregnancy

**DOI:** 10.1002/eji.200636542

**Published:** 2007-04

**Authors:** Pascale Kropf, David Baud, Sara E Marshall, Markus Munder, Angelina Mosley, José M Fuentes, Charles R M Bangham, Graham P Taylor, Shanti Herath, Beak-San Choi, Germán Soler, Tg Teoh, Manuel Modolell, Ingrid Müller

**Affiliations:** 1 Department of Immunology, Faculty of Medicine, Imperial College LondonLondon, UK; 2 Department of Obstetrics and Gynaecology, St. Mary's HospitalLondon, UK; 3 Department of Hematology, Oncology, and Rheumatology, University Hospital HeidelbergHeidelberg, Germany; 4 Departamento de Bioquímica y Biología Molecular, E.U. Enfermería y T.O., Universidad de ExtremaduraCáceres, Spain; 5 Department of Genito-Urinary Medicine and Communicable Diseases, Imperial CollegeLondon, UK; 6 Department of Veterinary Clinical Sciences, Royal Veterinary CollegeLondon, UK; 7 Departamento de Bioquímica y Biología Molecular, Facultad de Veterinaria, Universidad de ExtremaduraCáceres, Spain; 8 Department of Cellular Immunology, Max-Planck-Institute for ImmunobiologyFreiburg, Germany

**Keywords:** Arginase, l-Arginine metabolism, T cells, Tolerance/suppression

## Abstract

Complex regulation of T cell functions during pregnancy is required to ensure materno-fetal tolerance. Here we reveal a novel pathway for the temporary suppression of maternal T cell responses in uncomplicated human pregnancies. Our results show that arginase activity is significantly increased in the peripheral blood of pregnant women and remarkably high arginase activities are expressed in term placentae. High enzymatic activity results in high turnover of its substrate l-arginine and concomitant reduction of this amino acid in the microenvironment. Amino acid deprivation is emerging as a regulatory pathway of lymphocyte responses and we assessed the consequences of this enhanced arginase activity on T cell responses. Arginase-mediated l-arginine depletion induces down-regulation of CD3ζ, the main signalling chain of the TCR, and functional T cell hyporesponsiveness. Importantly, this arginase-mediated T cell suppression was reversible, as inhibition of arginase activity or addition of exogenous l-arginine restored CD3ζ chain expression and T cell proliferation. Thus, l-arginine metabolism constitutes a novel physiological mechanism contributing to the temporary suppression of the maternal immune response during human pregnancy.

## Introduction

The success of pregnancies poses an unresolved immunological challenge: the survival of the semi-allogeneic fetus *in utero* is critically dependent upon the induction of unresponsiveness of the maternal immune system, while the ability to respond to pathogens and other antigenic challenges needs to be retained. The mechanisms underlying immune tolerance during pregnancy are not fully understood. Over 50 years ago, Medawar [1] proposed three potential mechanisms that contribute to immunological unresponsiveness of the mother to the fetus, anatomic separation, antigenic immaturity of the fetus, and suppression or modification of the maternal immune system during pregnancy. Much progress has been made in the understanding of the immune mechanisms that prevent rejection of the fetus; however, this process is still not entirely elucidated in humans. Some of the salient features of the complex relationship between the maternal immune system and the fetus are starting to be understood, and it is accepted that multiple mechanisms provided by both the mother and the fetus contribute to the development and maintenance of tolerance and immune privilege [;^2^]. T lymphocytes can acquire tolerance in the thymus and in the periphery. Peripheral tolerance accounts for the “unresponsiveness” of T cells to tissue that express unique antigens. The placenta uniquely expresses allogeneic antigens and there is evidence that control or tolerization of anti-fetal T cells is critical [3]. It is clear that the maternal immune system is aware of fetal alloantigens during gestation, but is functionally tolerant of them until shortly after parturition [4]. Thus, transient suppression of the maternal immune response is necessary for fetal survival, and it is likely that several interconnected mechanisms have evolved to prevent rejection of the fetus. Recently, the inhibitory T cell costimulatory molecule programmed cell death ligand 1 has been shown to play a critical role in preventing maternal immune responses against the fetus and increasing apoptosis of T cells was considered as a potential mechanism [5]. A role for Fas-FasL, HLA-G or TRAIL-TRAIL-R in the apoptosis of maternal leukocytes during pregnancy has been shown in humans and in animal models [6–8]. Furthermore, regulatory T cells have also been implicated in the maintenance of tolerance and the prevention of maternal allogeneic responses against the fetus [9–11]. Indoleamine 2,3 dioxgenase (IDO), a tryptophan-catabolizing enzyme expressed in macrophages, dendritic cells and extravillous trophoblasts has also been identified as one of the mechanisms that plays a role in induction of tolerance and maintenance of allogeneic pregnancies [12–14]. Despite the acceptance that pregnancy is associated with suppression of cell-mediated immune responses, the mechanisms regulating this suppression are not well defined [15]. In the work we present here we have identified a novel pathway for the temporary suppression of immune responses in normal pregnancies.

The immunoregulatory function of l-arginine and of arginase, one of its metabolising enzymes, is increasingly recognized [16–20]. Modulation of T cell responses by arginase-induced l-arginine depletion is emerging as an important immunoregulatory pathway as demonstrated *in vitro* [21, 22], in animal models of infection [23–25], and in tumour evasion in humans [17–19]. l-Arginine is a semi-essential amino acid, *i.e*., it can be synthesized by adult humans, but must be supplemented by diet at times of physiological or pathological stress, such as pregnancy and fetal development [26]. It plays an essential role in protein synthesis and ammonia detoxification. Arginase, the final enzyme in the urea cycle, is responsible for the hydrolysis of l-arginine into urea and ornithine, which are further metabolized to proline or polyamines [27]. Two arginase isoforms exist (I and II), and these differ in subcellular localization, regulation and function [28]. It has been recently shown that during immune responses, consumption of l-arginine by arginase reduces the bioavailability of this amino acid in the microenvironment; this depletion of l-arginine induces down-regulation of CD3ζ chain expression in activated T cells [20, 29]. Down-regulation of CD3ζ expression results in uncoupling of the TCR signal transduction pathways and functional T cell hyporesponsiveness [30].

In the present study we investigated the expression and activity of arginase in human pregnancies, and determined the functional consequences of placental arginase on T cell responses. Our data show that arginase activity is enhanced in normal term pregnancy and that the activity of this enzyme is one of the mechanisms contributing to the suppression of maternal immune responses by reducing the bioavailability of l-arginine and leading to down-regulation of CD3ζ expression and induction of functional T cell hyporesponsiveness.

## Results

### Enhanced arginase expression in pregnancy

**Figure 1:**
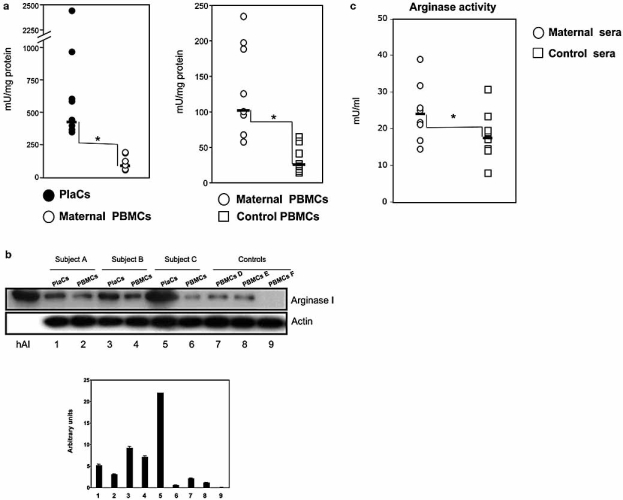
Arginase is enhanced in pregnancy. (a) PlaC (*n*=9) and maternal PBMC (*n*=9) were isolated and arginase activity was measured in cell lysates (left panel). Arginase activities in maternal PBMC were compared to those found in PBMC from age-matched non-pregnant controls (right panel). Each symbol represents arginase activity of the cells from one individual and the horizontal bar represents the median. Statistical differences were determined using the Mann-Whitney test: left panel *p<0.0001, right panel *p=0.008. (b) Arginase protein expression was measured in cell lysates (30 μg/sample) from paired individuals and from controls by Western blot as previously described [24, 53]. hAI: recombinant human arginase I. (c) Arginase activity was measured in maternal (*n*=9; open circles) and non-pregnant control sera (*n*=9; open squares). Each symbol represents the urea content in the serum of one individual and the horizontal bar represents the median. Statistical differences were determined using the Mann-Whitney test: *p=0.006.

Arginase has been purified from human placenta [31], but its role has not been characterized. We hypothesized that placental arginase induces T cell hyporesponsiveness, and that this is one of the mechanisms causing suppression of the maternal immune system in pregnancy. To test our hypothesis, we first measured arginase activity in cells isolated from human placenta (PlaC) immediately after parturition, and compared it with arginase activity in maternal peripheral blood mononuclear cells (PBMC) and PBMC from age-matched female non-pregnant controls. In all cases, arginase activity in PlaC was significantly higher than in paired PBMC (Fig. 1a, left panel). Although arginase activity in PBMC from pregnant women was notably lower than in PlaC, it was still significantly higher than in PBMC from controls (Fig. 1a, right panel). Determination of arginase protein by Western blot confirmed that higher arginase I protein expression was found in PlaC (Fig. 1b); however, arginase II expression was not detectable (data not shown). Sera prepared from maternal blood collected at the time of birth also contained significantly higher arginase activities than sera from controls (Fig. 1c), indicating an increased arginine turnover *in vivo*.

### Identification of the arginase-expressing cell type

**Figure 2:**
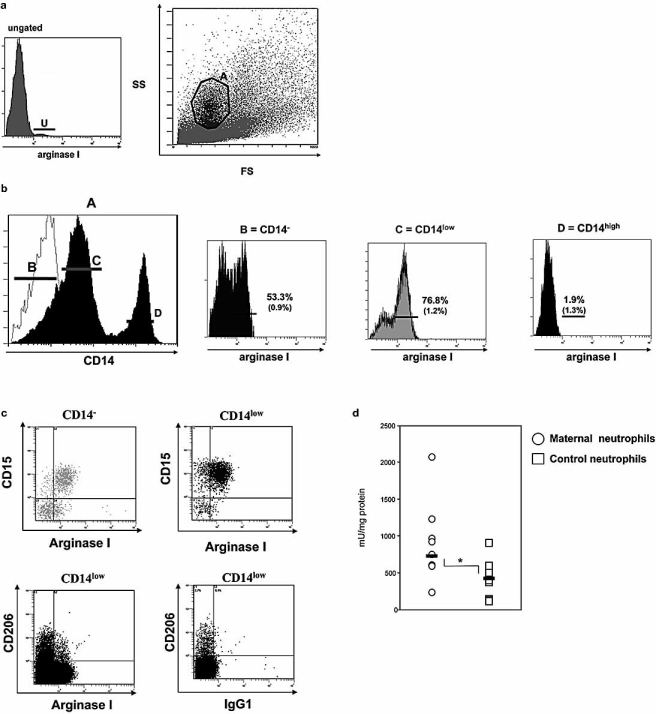
Identification of arginase expressing cells. (a) Identification of arginase I-expressing cells. The region containing cells expressing arginase I (A) was identified by back-gating ungated arginase I-positive cells (U) on the side scatter/forward scatter plot. (b) Frequency of arginase I-expressing cells. In region A, two populations of CD14^+^ cells were identified: CD14^low^ (C) and CD14^high^ (D)(CD14^–^: B, dotted line: unstained cells). The frequency of arginase-expressing cells was determined in region B (CD14^–^), C (CD14^low^) and D (CD14^high^). The markers of histograms and dot-plot were set according to the fluorescence of unstained cells and the value in parenthesis represents the percentage of cells positive for the isotype control. Data show the results of one representative experiment out of four independent experiments. (c) Neutrophils and alternatively activated macrophages express arginase I. Arginase I was expressed in 77.8% of CD15^+^CD14^–^ cells (upper left), 90.8% of CD15^+^CD14^low^ cells (upper right) and 25.3% of CD14^low^CD206^+^ cells (lower left); the isotype control for arginase I in CD14^low^CD206^+^ is shown in the lower right (1.7%). Data show the results of one representative experiment out of four independent experiments. (d) Arginase activity in peripheral neutrophils is increased during pregnancy. Neutrophils were isolated by double-density gradient centrifugation of Histopaque®-1119 and 1077 and arginase activity was measured as described in *Material and methods*. Each symbol represents arginase activity of the cells from one individual and the horizontal bar represents the median. Statistical differences were determined using the Mann-Whitney test: *p=0.02.

The type of arginase-expressing cells in the placenta was determined by a combination of intracellular protein staining and cell surface labelling. Arginase-expressing cells were identified in region A (forward scatter/side scatter plot, Fig. 2a) and were contained in the CD14^–^ and CD14^low^ populations (Fig. 2b, regions B and C); monocytic CD14^high^ cells did not express arginase I (Fig. 2b, region D). The majority of arginase-expressing cells were neutrophils (CD15^+^CD14^–^ and CD15^+^CD14^low^, Fig. 2c, upper left and upper right dot plots). Another small population of arginase-expressing cells was identified within the CD14^low^ population. These were alternatively activated macrophages, as characterized by the co-expression of the mannose receptor CD206 (Fig. 2c, lower left dot plot). Thus, two populations of cells express arginase in suspensions obtained from placental biopsies: neutrophils and alternatively activated macrophages.

Neutrophils are the main arginase-expressing cells in the placental biopsies; however, it is not known whether arginase activity in neutrophils isolated from peripheral blood of pregnant women is higher than in non-pregnant controls. To answer this question, we isolated neutrophils from blood obtained at the time of delivery and compared it to neutrophils isolated from the peripheral blood of non-pregnant controls. The results in Fig. 2d clearly demonstrate that arginase activity was significantly higher in peripheral blood neutrophils from pregnant women. Similar results were obtained by flow cytometry by comparing the mean fluorescence intensities (MFI) of arginase I expression in peripheral blood neutrophils from pregnant women with those from non-pregnant controls (2.1 ± 0.3 *vs.* 1.3 ± 0.1, respectively). The MFI of arginase I expression of neutrophils isolated from paired placental biopsies and peripheral blood of the same pregnant women were similar (1.9 ± 0.4 *vs.* 2.1 ± 0.3, respectively).

### Determination of the functional consequences of placental arginase activity

**Figure 3:**
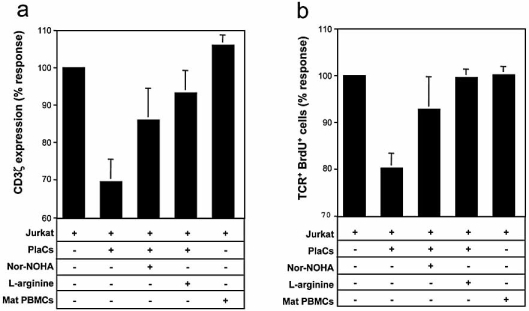
PlaC have the capacity to down-regulate CD3ζ chain and suppress proliferation of Jurkat cells in an arginase-/l-arginine-dependent manner. PlaC or maternal PBMC (2 × 10^5^ cells) were incubated with 1 × 10^5^ Jurkat cells in a final volume of 200 μL in the presence or in the absence of 10 μL 5.6 μM nor-NOHA or 2 μL 100 mM l-arginine. After 2 days, MFI of CD3ζ in TCR^+^ cells (a) or frequency of TCR^+^ cells incorporating BrdU (b) were determined. Jurkat cells were identified by increased side /forward scatter. A 100% response represents the value of the MFI of CD3ζ (a: MFI =4.6) or the percentage of proliferating Jurkat cells (b: 36.3%) in the cultures containing Jurkat cells alone. Data show the average ± SEM of three independent experiments.

To examine the functional effect of placental arginase, PlaC and Jurkat cells were co-cultured to assay arginase-induced modulation of T cell responsiveness [21, 29]. Co-culture of PlaC with Jurkat cells resulted in both down-regulation of CD3ζ chain expression (Fig. 3a) and decreased proliferation of Jurkat cells (Fig. 3b). Importantly, CD3ζ chain expression and T cell proliferation could be restored by addition of either a competitive arginase inhibitor, *N*^ω^-hydroxy-nor-l-arginine (nor-NOHA [32]) or by the addition of exogenous l-arginine (Fig. 3a, b). In contrast, maternal PBMC had no effect on CD3ζ chain expression or proliferation of Jurkat cells. These results demonstrate that PlaC can mediate CD3ζ chain down-regulation and T cell hyporesponsiveness through arginase-mediated l-arginine depletion.

### CD3ζ down-regulation in TCR^+^ cells from placental biopsies is reversible by modulating l-arginine metabolism

**Figure 4:**
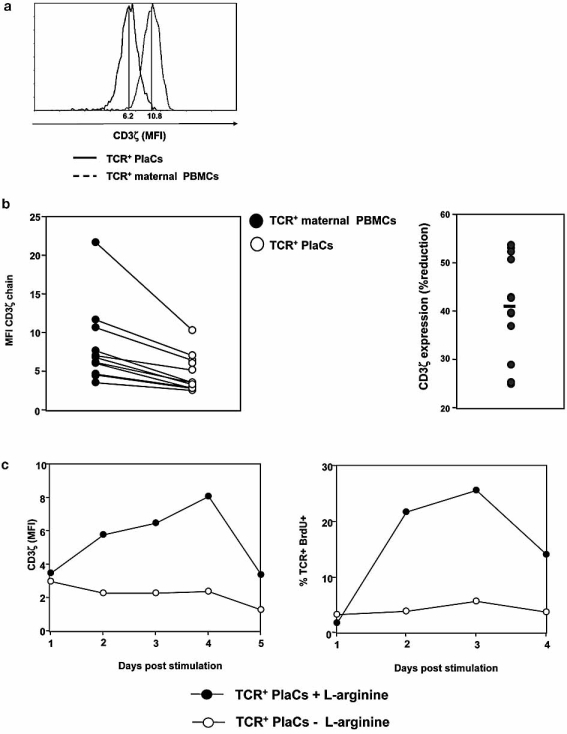
Down-regulation of CD3ζ chain in T cells from placental biopsies. (a) MFI of CD3ζ chain expression was determined in maternal TCR^+^ PBMC (dotted line) and in TCR^+^ PlaC (solid line). The data show the result of 1 representative experiment out of 13 independent experiments. (b) Each symbol represents the MFI of CD3ζ in peripheral TCR^+^ cells (closed circles) and paired TCR^+^ cells from placental biopsies (open circles) from one individual (left panel, *n*=13). Percent reduction in CD3ζ MFI in TCR^+^ cells from PlaC as compared to TCR^+^ cells from maternal PBMC (right panel). (c) Down-regulation of CD3ζ chain and proliferation of TCR^+^ PlaC is reversible. PlaC (4 × 10^5^) were stimulated with plate-bound anti-CD3 and anti-CD28 mAb in a final volume of 200 μL in complete DMEM in the absence of l-arginine, or in complete DMEM containing 0.1, 0.4, 1 or 2 mM l-arginine. At the indicated times post stimulation, cells were harvested and the MFI of CD3ζ in TCR^+^ cells (left panel) or the frequency of TCR^+^ cells incorporating BrdU (right panel) were determined. Similar results were obtained with all the concentrations of l-arginine tested and the data show the results obtained with 1 mM. Data show the results of one representative experiment out of four independent experiments.

To determine the functional consequences of local arginase activity on T cells within the placenta, we examined the expression of CD3ζ in TCR^+^ cells in placental cell suspensions directly *ex vivo*. In all individuals examined, CD3ζ chain expression was significantly reduced in TCR^+^ cells derived from placental biopsies as compared to maternal T lymphocytes in peripheral blood (average reduction: 40.8 ± 2.8%) (Fig. 4a, b). These results demonstrate that CD3ζ chain expression is down-regulated in TCR^+^ cells in the placenta, a compartment with high arginase activity. Since TCR^+^ cells circulate between placenta and periphery, we speculated that this down-regulation would be reversible, to allow the maternal immune system to respond to other antigenic challenges whilst maintaining a state of non-responsiveness against the fetus. To test this, we assessed whether CD3ζ down-regulation in TCR^+^ cells from placental tissues could be reversed. In the presence of l-arginine, TCR-mediated stimulation of PlaC resulted in increased CD3ζ chain expression in TCR^+^ cells; in the absence of exogenous l-arginine no recovery was observed (Fig. 4c, left panel). This shows that CD3ζ chain down-regulation in TCR^+^ cells in placental tissues is reversible. A functional correlate of CD3ζ chain expression is the ability of cells to proliferate. TCR^+^ cells obtained from placental tissues and stimulated in the presence of l-arginine retain this ability (Fig. 4c, right panel). These experiments demonstrate that the functional phenotype of TCR^+^ cells from placental biopsies is reversible and that the reduced responsiveness of these T cells cannot be due to apoptosis.

### Impact of placental arginase on autologous peripheral T cells

**Figure 5:**
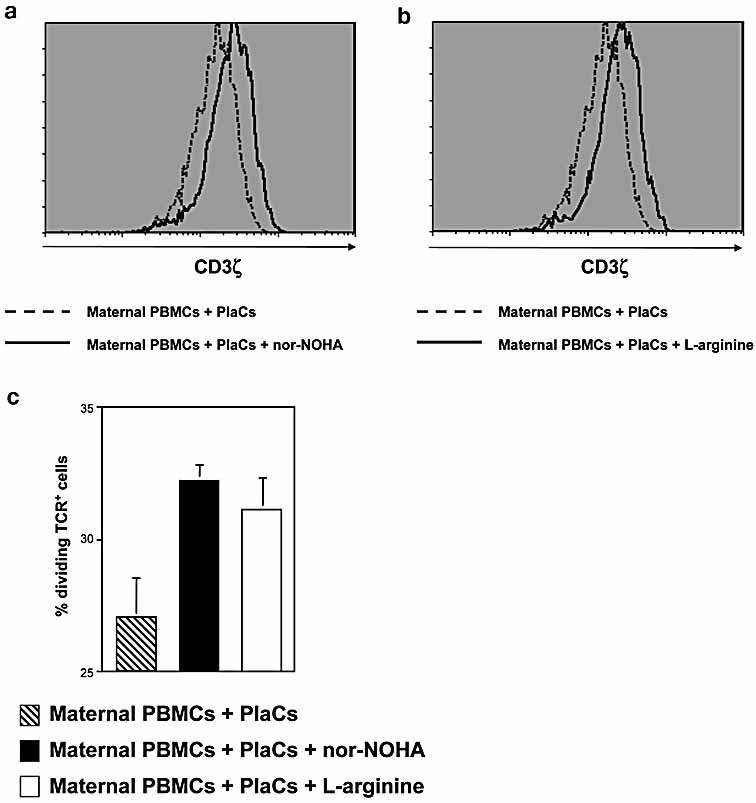
Down-regulation of CD3ζ chain and proliferation by PlaC is restored by inhibition of arginase or by addition of l-arginine. PlaC (2 × 10^5^) were stimulated with plate-bound anti-CD3 mAb and anti-CD28 mAb in a final volume of 100 μl DMEM (0.1 mM l-arginine). Then 10 μL nor-NOHA (5.6 μM) was added to some of the wells, and 30 min later maternal PBMC (1 × 10^5^, labeled with CFSE to differentiate maternal TCR^+^ from placental TCR^+^) were added in a final volume of 100 μL. l-Arginine (2 μL 100 mM) was added to some of the wells. (a, b) After 2 days, the cells were harvested and the expression of CD3ζ was determined in maternal TCR^+^ cells (CFSE^+^ cells). Data show the results of one representative experiment out of three independent experiments. (c) The frequency of cells having undergone at least one cell division was determined in maternal TCR^+^ CFSE^+^ cells. Data show the results ± SD of one representative experiment out of three independent experiments.

Finally, to confirm the role of placental arginase and l-arginine in mediating T cell hyporesponsiveness, autologous maternal PBMC were stimulated in the presence of PlaC, and the effects of arginase inhibition or addition of exogenous l-arginine on maternal TCR^+^ cells were assessed. Competitive inhibition of arginase (nor-NOHA) resulted in a clear up-regulation of CD3ζ chain in maternal TCR^+^ cells (Fig. 5a); similarly, addition of exogenous l-arginine also enhanced CD3ζ expression (Fig. 5b). In agreement with these data, the proliferative response of maternal TCR^+^ cells was also increased in the presence of nor-NOHA or exogenous l-arginine (Fig. 5c). These results demonstrate that PlaC can mediate local and reversible T cell hyporesponsiveness in the placenta, directly linking arginase and the bioavailability of l-arginine to feto-maternal tolerance.

## Discussion

The survival of the semi-allogeneic fetus is critically dependent upon the induction of unresponsiveness of the maternal immune system. Here we reveal a physiological mechanism whereby arginase can mediate suppression of maternal T cell responses. We show that high arginase activity is present in cells isolated from placenta, and that the source of arginase is mainly neutrophils and a population of alternatively activated macrophages. It has to be noted that arginase levels in cells obtained from placental biopsies are comparable to those in the liver, the organ with the highest arginase concentration in the human body [28]. High arginase levels in the placenta imply a high rate of substrate consumption and decreased levels of extracellular l-arginine. This is supported by down-regulation of CD3ζ chain expression in TCR^+^ PlaC, a finding consistently associated with l-arginine depletion [20, 21, 29]. Although T cell hyporesponsiveness associated with down-regulated CD3ζ chain has been observed in a variety of disease manifestation, the molecular mechanisms leading to the down-regulation of one of the main the signalling chains of the TCR and the associated immune suppression are not well defined. We show here that the T cell hyporesponsiveness in human pregnancy is reversible and that T cells in the placenta retain their capacity to respond to stimulation. In addition, PlaC co-cultured with peripheral blood lymphocytes induce arginase-dependent, l-arginine mediated T cell hyporesponsiveness, indicating a novel pathway through which immune privilege can be mediated at the feto-maternal interface.

In accordance with our finding that the majority of arginase-expressing cells in the cell population obtained from placental biopsies are neutrophils, some authors of the current study have recently identified human granulocytes, the largest subpopulation of myeloid cells, as the main subset of arginase I-expressing cell. They demonstrated that arginase I, which is localized in azurophil granules of these cells is a novel fungicidal effector pathway [33]. Moreover, arginase from granulocytes of patients with purulent inflammation has immunosuppressive effector functions; it induces profound suppression of T cell proliferation and cytokine synthesis *via* arginase-mediated arginine depletion [34]. The anti-inflammatory function of arginase-expressing human neutrophils supports the concept that neutrophils can not only initiate but also contribute to the resolution of inflammation. Neutrophils are one of the key cell types contributing to innate and adaptive immune responses and it is known that changes in their functional properties occur during pregnancy [33, 35, 36]. The placenta is a likely site of immunoregulation and indeed, trophoblasts have been shown to regulate the activation status of neutrophils [37]. Proinflammatory responses and placental oxidative damage have been associated with pregnancy complications such as preeclampsia, miscarriage and preterm labor [38, 39]. Thus, human granulocyte arginase constitutes a novel homeostatic immunoregulatory mechanism that limits inadequate or excessive immune activation.

l-Arginine metabolism is emerging as an important regulator of T cell responses in humans [20]. Arginase-expressing polymorphonuclear granulocytes in renal cell carcinoma patients have been shown to impair T cell responses through depletion of l-arginine in the extracellular environment 17], and inhibition of arginase has been shown to boost anti-tumour T cell responses, and reverses the anergic state of tumour-infiltrating T lymphocytes in human prostate cancers 18]. In patients with active pulmonary tuberculosis, a correlation between T cell hyporesponsiveness and arginase-mediated l-arginine depletion has also been described [40]. Interestingly, and in accordance with the findings we report here, the T cell hyporesponsiveness was reversible: T cells of patients that responded to anti-tuberculosis therapy re-expressed the signal transducing proteins [40]. l-Arginine has been shown to play a role in the regulation of mRNA translation [41, 42], and, while the exact mechanism through which it mediates T cell unresponsiveness is unclear, it is likely that reduced protein synthesis contributes to this effect.

Immunoregulation *via* arginase-mediated l-arginine depletion has similarities with the well-characterized effects of IDO-mediated tryptophan degradation. IDO exerts its antimicrobial effector functions *via* the depletion of amino acid essential for microbial growth [43] and it suppresses immune responses. The tryptophan metabolism plays an analogous role in regulation of maternal immune responses in the mouse and pioneering work has revealed that placental cells expressing IDO induce the catabolism of tryptophan, which results in suppression of maternal T cell activation and prevents rejection of allogeneic fetuses [12]. In the mouse, limiting tryptophan availability appears to be an important mechanism by which the local microenvironment can influence the outcome of T cell activation [13]. Interestingly, in contrast to the striking effects of chemical inhibition of IDO in allogeneic pregnancies, genetic elimination of IDO did not affect fetal viability and pregnancy success rates. These studies indicate that alternative compensatory or redundant mechanisms prevent fetal rejection [44]. We have identified another physiological pathway, the arginase induced metabolism of l-arginine that results in T cell hyporesponsiveness in normal human pregnancies. Similarly to the reported discrepancies in the IDO model, inhibition of arginase activity *in vivo* using chemical inhibitors affects the reproductive process in a rat model [45]. A later study confirmed the anti-fertility effects of blocking arginase activity and showed that it acts mainly by inhibiting implantation or by increasing resorption of implants [46]. Inactivation of the arginase gene, however, did not affect pregnancy success since heterozygous arginase I-knockout mice produced the statistically expected rate of homozygous arginase-deficient offspring [47], which died within 2 weeks after birth of hyperammonemia. It is likely that both IDO and arginase reduce the bioavailability of amino acids and exert their immunomodulatory function using similar pathways to silence T cells. An important distinction between immune hyporesponsiveness mediated by IDO and that induced by arginase is that the former results in apoptosis of T cells [48], whereas the hyporesponsiveness induced by placental arginase is reversible. Immunoregulation by amino acid availability has also been reported for cysteine. Cysteine depletion leads to cell cycle arrest in human T cells due to intracellular glutathione reduction and altered redox regulation [49].

Our results strengthen the evidence for the immunoregulatory potential of amino acid metabolism. Arginase-mediated T cell hyporesponsiveness is not only relevant for the understanding of suppression of maternal immune responses during pregnancy, it has wider implications and will contribute to the understanding of peripheral tolerance or unresponsiveness in a wide range of infectious diseases and pathologies. Modulation of T cell responses by these metabolic pathways will open new avenues of therapeutic intervention for a wide range of pathological conditions.

## Materials and methods

### Subjects

The study protocol was approved by the local Ethics Committee of St. Mary's NHS Trust, London (COREC reference 05/Q0403). Twenty-six pregnant women (mean age 31.0 ± 6.0 years) were recruited at the time of uncomplicated spontaneous vaginal delivery or elective caesarian section. Exclusion criteria included any major complication of pregnancy or intercurrent illness, such as pre-eclampsia, pre-or post-term labor (<38 weeks or >42 weeks), intra-uterine growth retardation, gestational diabetes, or viral or parasitic infections. No major differences were observed in cells isolated from placentae obtained from spontaneous vaginal delivery or by caesarian section.

Twenty-one healthy, non-pregnant women (mean age 29.6 ± 6.1 years) were recruited as controls. All subjects gave written, informed consent before participation.

### Samples

Blood was collected in EDTA tubes, PBMC were isolated by density gradient centrifugation on Histopaque®-1077 and neutrophils by double density gradient centrifugation on Histopaque®-1119 and Histopaque®-1077. Cells were washed and resuspended in PBS until further use.

Whole placentae were harvested directly after parturition and biopsies were taken through the full thickness of the placenta. Single cell suspensions were obtained by homogenising biopsies in PBS on cell dissociation sieves; a Histopaque gradient was performed to eliminate debris and the cells obtained from these placental biopsies (PlaC) were washed and resuspended in PBS until further use. Granulocytes (<3%) are virtually excluded from purified PBMC from healthy donors; their sedimentation with PlaC is likely to be due to *in vivo* activation since it has been shown that receptor-mediated granulocyte activation leads to changes in their density [50].

### Arginase activity

The enzymatic activity of arginase was measured as previously described [24, 33, 51]. Briefly, cells were lysed with 100 μL of 0.1% Triton X-100. After 30 min on a shaker, 100 μL 25 mM Tris-HCl was added. To 100 μL of this lysate, 10 μL 10 mM MnCl_2_ was added, and the enzyme was activated by heating for 10 min at 56°C. Arginine hydrolysis was conducted by incubating the lysate with 100 μL 0.5 M l-arginine (pH 9.7) at 37°C for 15–20 min. The reaction was stopped with 800 μL H_2_SO_4_ (96%)/H_3_PO_4_ (85%)/H_2_O (1:3:7, v/v/v). The urea concentration was measured at 540 nm after addition of 40 μL α-isonitrosopropiophenone (dissolved in 100% ethanol) followed by heating at 95°C for 30 min. To determine arginase activity in the sera, urea concentrations were first determined in the sera, without the activation and hydrolysis steps; these values were subtracted from those obtained by measuring the urea levels as described above.

One unit of enzyme activity is defined as the amount of enzyme that catalyzes the formation of 1 μmol of urea per min.

### Western blot analysis

Arginase I and II protein expression were measured as described previously [33, 52, 53]. Equal amounts of protein (30 μg/condition) from PBMC and PlaC were resolved by SDS-12% gel electrophoresis and transferred to polyvinylidene difluoride (PVDF) membranes. Briefly, proteins were transferred (250 mA for 60 min) to PVDF membranes using a Mini Trans-Blot Cell apparatus (Bio-Rad). The procedure for immunodetection includes transfer, blocking of the membrane (30 min at 37°C) with 10 mM Tris-HCl pH 7.5, 150 mM NaCl and 0.2% Tween-20 (TTBS) containing 10% non-fat dried milk and incubating (60 min at room temperature) with the primary antibody (anti-arginase I rabbit polyclonal antibody [24] and anti-arginase II rabbit polyclonal IgG, Santa Cruz Biotechnology, were used to detect arginase). After washing (two times for 5 min with TTBS), membranes were incubated (60 min at room temperature) with peroxidase-conjugated secondary antibodies (1:5000 in TTBS with 10% non-fat dried milk). After washing (two times for 5 min and once for 10 min), detection of bound antibodies was visualized by chemiluminescence using the ECL^TM^-plus reagent (Amersham). Semi-quantification of was performed by enhanced chemiluminescence. The signal was detected by GelDoc system (Bio-Rad^©^) and quantified with Quantity-One-1D analysis software (Bio-Rad^©^). Human arginase I was a generous gift from Dr. S. D. Cederbaum.

### Flow cytometric analysis

Antibodies used were as follows: anti-CD3ζ (Santa Cruz: clone 6B10.2), anti-CD14 (BD Pharmingen: cloneM5E2, Beckman Coulter: RMO52), anti-CD206 (BD Pharmingen: clone 19.2), anti-TCR (BD Pharmingen: clone T10B9.1A-31, eBioscience: clone IP26), anti-BrdU (with DNase, BD Pharmingen: clone B44); anti-arginase I (HyCult Biotechnology: clone 6G3) and the isotype control (BD Pharmingen: clone MOPC21) were coupled with Alexa Fluor^R^ 488 (Molecular Probes). BrdU (BD Pharmingen) and CFSE (Molecular Probes) were added at a final concentration of 10 μM and 0.4 mM, respectively. BrdU was added during the last 4 h of culture. Cells were washed with PBS, the fixation step was performed with 2% formaldehyde in PBS and the permeabilisation step with 0.5% saponin in PBS. For the intracellular detection of arginase I, 1 × 10^6^ PlaC were incubated with 20 μL FcR blocking reagent for 20 min at room temperature. Anti-CD14, anti-CD15 and anti-CD206 mAb were added directly to the tubes. After 20 min at 4°C, the cells were washed, fixed and permeabilised and resuspended in 200 μL PBS/5% FBS; 20 μL FcR blocking reagent was added to the cells for 5 min. Then 0.2 μg FITC-conjugated anti-arginase I or mouse FITC-conjugated IgG1 was added to the cells, and 20 min later the cells were washed. The cells were analysed with an EPICS XL instrument (Beckman Coulter).

### Cell culture

PlaC, maternal PBMC and control PBMC were stimulated with plate-bound anti-CD3 mAb (30 ng, Serotec: clone UCHT1) and anti-CD28 mAb (3 ng, Serotec: clone YTH913.12).

Dulbecco's modified essential medium (DMEM, 0.4 mM l-arginine unless stated otherwise) and DMEM without l-arginine (Gibco) were supplemented with 50 IU/mL penicillin, 50 μg/mL Streptomycin, 292 μg/mL l-glutamine and 5% FBS.

### Statistical analyses

Data were evaluated using the Mann-Whitney test with GraphPad PRISM version 2.0.

## Abbreviations

IDO: Indoleamine 2,3 dioxgenase: **nor-NOHA:** *N*^ω^-hydroxy-nor-l-arginine
PlaC: human placenta cells
